# Topiramate-Induced Coma in a 39-Year-Old Female Patient: Burst Suppression on EEG

**DOI:** 10.7759/cureus.39127

**Published:** 2023-05-17

**Authors:** Abdullah M Hakoun

**Affiliations:** 1 Department of Neurology, Saint Louis University School of Medicine, St. Louis, USA

**Keywords:** burst, seizure, non-anion gap metabolic acidosis, coma, topiramate

## Abstract

Reported cases of topiramate ingestion resulting in coma and generalized convulsive status epilepticus are very rare. Such a phenomenon of a relatively safe antiepileptic drug (AED) causing serious neurological compromise should be carefully reviewed. A 39-year-old female with a history of uncontrolled epilepsy, migraine headaches, hypothyroidism, obsessive-convulsive disorder, and depression presented with generalized tonic-clonic seizures that progressed to status epilepticus and coma thereafter. She was intubated due to a depressed level of consciousness and transferred afterward to our hospital. Electroencephalography (EEG) demonstrated a burst suppression pattern without receiving any sedating agents. The level of consciousness improved on the fourth day, and she achieved complete neurological recovery by the sixth day of hospitalization. She was offered AEDs and supportive therapy during her admission. Upon further investigation into the cause of her seizures, it was discovered that she had ingested a large dose of topiramate in a suicide attempt.

## Introduction

Topiramate has been used in the management of various neurological disorders since 1996. It has a therapeutic dose of 25-800 mg per day in the serum and a relatively safe clinical profile [1]. However, in certain instances, it can lead to complications and deleterious consequences. The most common side effects of topiramate include dizziness, fatigue, visual disturbances, ataxia, mental slowing, and impaired concentration [2]. Anhidrosis and hyperthermia leading to heat stroke have also been reported in rare circumstances [3]. Topiramate-induced coma followed by generalized convulsive status epilepticus is truly rare and has only been reported in two patients in the past [4]. We review a case of a 39-year-old female admitted for a coma following a spell of tonic-clonic convulsions.

## Case presentation

A 39-year-old female was taken to a neighboring hospital after being found unresponsive at home following an episode of a convulsive seizure. She had a past medical history significant for obsessive-compulsive disorder, depression, anxiety, migraine headaches, syncopal events, thyroid cancer that was resected and complicated by hypothyroidism, and uncontrolled epilepsy. She progressed to convulsive status epilepticus and was treated with antiepileptic drugs (AEDs) and subsequently transferred to a tertiary care facility. Upon arrival at the Neurocritical Care Unit (NCCU), the patient had a blood pressure of 88/71 mmHg. Temperature was noted at 35.5°Celsius (95.9°Fahrenheit), heart rate at 67 beats per minute, respiratory rate at 16 breaths per minute, and oxygen saturation at 100% on room air, with a Glasgow Coma Scale (GCS) of 6 (E1V1M4). Neurological examination was remarkable for pupils that are 3 mm in diameter and minimally reactive to light, absence of cough, gag, and corneal reflexes while withdrawing all extremities in response to noxious stimuli.

She underwent endotracheal intubation and mechanical ventilation secondary to a depressed level of consciousness with a GCS < 8. Initial arterial blood gas analysis showed a mild non-anion gap metabolic acidosis (NAGMA) with respiratory compensation (pH: 7.34, PaCO2: 27.4, PaO2: 98.2, HCO3: 14), lactate of 0.6 mmol/L, sodium of 145 mmol/L, potassium of 3.3 mmol/L, chloride of 120 mmol/L, glucose of 175 mg/dL, and an anion gap of 11. Central and arterial lines were placed for strict blood pressure monitoring and resuscitation. She received 2 L of crystalloid intravenous fluids followed by starting norepinephrine for blood pressure augmentation. Urine and serum drug screening panels were both unremarkable for cocaine and metabolites, opiates, barbiturates, oxycodone, methadone, amphetamines, methamphetamines, benzodiazepines, buprenorphine, phencyclidine, and cannabinoids. The patient received 4 mg of lorazepam and a fosphenytoin loading dose of 1500 mg after experiencing additional tonic-clonic seizures in the NCCU. Continuous electroencephalography (EEG) was initiated and consisted of discontinuous patterns with periods of generalized suppression alternating with periods of high-amplitude bursts of mixed slow and fast frequencies and sharp waves. MRI of the brain with and without contrast revealed an incidental arachnoid cyst, without any acute intracranial abnormality or findings to suggest a structural etiology for her seizures. The patient had a lumbar puncture with cerebrospinal fluid (CSF) analysis, which proved to be non-diagnostic (WBC count: 2/uL, RBC count: 0/uL, protein: 34 mg/dL, glucose: 81 mg/dL, gram stain and culture showed no growth or organisms). Fosphenytoin 100 mg every eight hours and levetiracetam 1,000 mg twice a day were initiated as maintenance doses. Upon further review of medical records, the patient was found to have been taking multiple medications at home, including baclofen, furosemide, levothyroxine sodium, naproxen, potassium chloride, and ibuprofen on an as-needed basis. Levothyroxine sodium 112 mcg was solely restarted.

**Figure 1:**
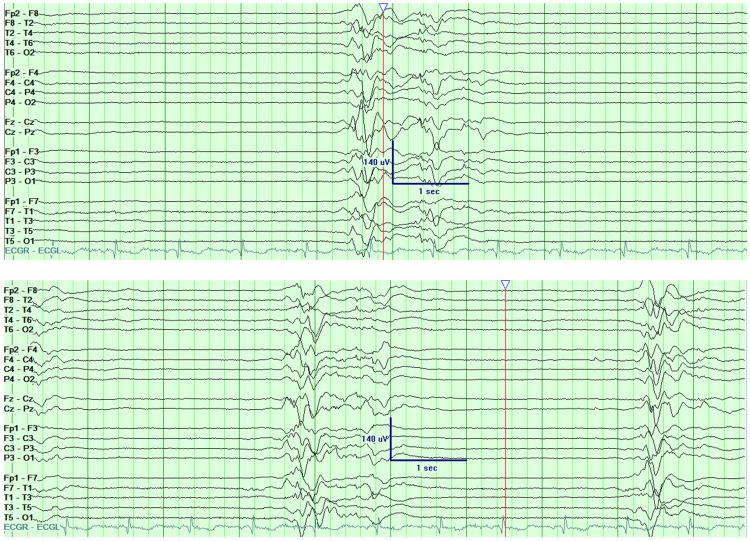
EEG showing 1-3 seconds of semi-rhythmic theta activity followed by suppression. The recording showed multiple inter-burst periods ranging from two to 10 seconds and with bursts lasting 1-3 seconds. Myoclonic jerking episodes were correlated with burst episodes, followed by periods of non-responsiveness during suppression. EEG: electroencephalography

The following day, the patient started experiencing myoclonic jerking and pupillary dilation without electrographic correlates on continuous EEG. Lorazepam 2 mg IV resulted in a temporary resolution of her myoclonus. Repeat arterial blood gas showed worsening NAGMA with a pH of 7.262. The patient’s level of consciousness continued to decline with a nadir GCS of 3 (E1V1M1) on the second day of admission. On the third day, the patient continued experiencing intermittent myoclonic activity throughout the day. However, EEG steadily improved from an initial discontinuous burst suppression pattern to generalized periodic epileptiform discharges (GPEDs), then to a predominant encephalopathic pattern with persistent epileptiform waves (Figure 1). She also had a return of cough, gag, and corneal reflexes and was making occasional semi-purposeful movements with her extremities. On the fourth day of admission, the patient’s family reported finding empty bottles of topiramate and levothyroxine sodium that were found in the patient’s room. Relevant serum levels for topiramate, thyroid-stimulating hormone (TSH), free thyroxine (FT4), and free triiodothyronine (FT3) were sent on that day. The topiramate level on the fourth day was 0.15 mg/dL (reference range: 0.5-2 mg/dL). TSH levels changed from 0.03 on the day of admission to 0.93 on the fourth day of hospitalization (reference range: 0.35-4.94 mU/L).

On the fifth day, the patient’s level of GCS score improved to 12 (E4V2M6), and her EEG showed an encephalopathic pattern consisting of high voltage, diffuse 1 Hz delta frequency with some intermittent alpha and theta frequencies. The patient was successfully liberated from the ventilator on the fifth day. On the sixth day, the patient had a GCS of 15 (E4V5M6) and was fully alert, oriented, and able to communicate. Her speech was clear and fluent with good repetition, comprehension, and naming. The remainder of the neurological examination was unremarkable.

## Discussion

Topiramate has been used in the management of various neurological disorders. It has a high therapeutic index and a relatively safe clinical profile. Topiramate toxicity has been reported when ingested alone in high doses or when taken in combination with other medications [5,6]. Topiramate side effects in the setting of therapeutic doses are mild; the most common are related to the central nervous system. These include paresthesia, somnolence, fatigue, dizziness, and mild cognitive and memory impairment [7]. Rarely, topiramate-induced anhidrosis and hyperthermia sometimes leading to heat stroke have been reported [3]. However, coma accompanied by myoclonic jerking or status epilepticus following topiramate ingestion is limited to three case reports and lasted for 12, 18, and 20 hours only [4,8,9]. Our patient remained comatose for 96 hours with a discontinuous EEG pattern while withholding iatrogenic sedation. Other case reports documented a burst suppression pattern seen with baclofen toxicity [10]. However, baclofen toxicity as a sole cause of this patient’s presentation would be unlikely given the lack of an associated kidney injury or respiratory metabolic acidosis. An argument can be made that there is a synergistic effect on the central nervous system when ingesting topiramate along with baclofen. Another aspect worth considering is the patient’s thyroid status. Elevated or low levels of thyroid hormone are known to cause significant effects on the neuroendocrine axis. However, other signs (e.g., hypothermia/hyperthermia, elevated blood pressure, bradycardia or tachycardia, and constipation or diarrhea) have been absent. Additionally, FT3 and FT4 levels were within normal limits.

The patient’s significant psychiatric history and access to neurotropic medications raised suspicion for toxic ingestion and potential overdose. Despite topiramate levels being within normal limits, it was obtained days after the ingestion. Additionally, a strong history suggesting ingestion was provided by the family after finding an empty bottle of recently filled topiramate. The review done by Lovinger et al. [11] suggests that topiramate can cause severe symptomatic NAGMA even when at therapeutic levels. NAGMA experienced during hospitalization (Table 1) resolved within two days of presentation and is consistent with topiramate activity against carbonic anhydrase CA-II and CA-IV isoenzymes [8,9]. The drug is eliminated unchanged mainly through kidney excretion. It is however negligibly bound to plasma proteins and is eliminated partly by oxidation and hydrolysis. In healthy individuals, the half-life is about 20-30 hours. However, the elimination rate can be accelerated in patients taking concomitant enzyme-inducing drugs such as phenytoin, carbamazepine, and barbiturates [2].

**Table 1:** Laboratory characteristics during days 1, 2, 3, and 4 of hospitalization.

Day of admission	Arterial pH (7.35-7.45)		PaCO2 (35-39 mmHg)	Serum HCO3- (19-25 mmol/L)		Serum Na+ (136-144 mmol/L)	Serum K+ (3.5-5 mmol/L)	Serum Cl- (102-110 mmol/L)	Anion gap (8-14 mmol/L)
1	7.343		27.4	14		145	3.3	120	11
2	7.262		31	15		147	3.7	123	7
3	7.361		34.3	18.9		146	3.5	120	5
4		7.409	37.3		23.1	143	3.3	113	7

Numerous cases exist in the literature describing the ingestion of large quantities of topiramate with a wide range of symptoms and severity. One examination of state poison control center records identified 56 patients with topiramate overdose, and 74% of these patients had no appreciable symptoms or only minor symptoms [12]. However, the average dose ingested in this study was only twice the daily recommended limit. One published case describes a 24-year-old female patient who ingested 4,000 mg in a suicide attempt yet remained completely asymptomatic with stable vitals [13]. Conversely, another report of a 21-year-old male who ingested 8,000 mg of topiramate presented with a generalized seizure pattern that was well controlled with lorazepam [14].

The literature generally reports positive outcomes in these patients, and supportive care is often sufficient. An examination of 29 case reports found that death occurred in only three patients who had ingested excessive topiramate (usually with other substances that may account for the lethality) [15]. Large-scale analyses of topiramate overdoses are not well described, and many reported incidents occur through case studies. An examination of state databases, such as that performed by Wills et al. [12], may help clarify these patients’ clinical progressions and potential complications.

To the best of our knowledge, this is the first reported case of topiramate toxicity demonstrating a discontinuous burst suppression pattern on EEG lasting 48 hours accompanied by convulsive status epilepticus.

## Conclusions

We report a rare case of intentional topiramate overdose leading to prolonged coma accompanied by generalized convulsive status epilepticus, myoclonic activity, burst suppression pattern on EEG, and NAGMA. Topiramate-induced coma and provoked seizures are reasonable considerations in patients with access to this medication, especially when taken in excess or in combination with other medications. A high index of suspicion should be maintained even with low or therapeutic levels of topiramate. Treatment of this condition is largely supportive with close observation, cardiopulmonary support, mechanical ventilation, vasopressors, correction of acid-base and electrolyte abnormalities, and aggressive treatment of seizures, possibly with fosphenytoin to aid in the metabolism and clearance of topiramate.
